# Plate augmentation and hybrid bone grafting are effective treatments for atrophic nonunion of the femur with the original intramedullary nail retained in situ

**DOI:** 10.1038/s41598-024-57809-1

**Published:** 2024-03-26

**Authors:** Huang Qiang, Zhang Congming, Ma Teng, Zhang Kun, Wang Chaofeng

**Affiliations:** Department of Orthopaedic Surgery, Hong Hui Hospital, Xi’an Jiaotong University College of Medicine, No.76 Nanguo Road, Beilin District, Xi’an, 710054 Shaanxi China

**Keywords:** Atrophic nonunion, Femur, Plate augmentation, Hybrid bone grafting, Intramedullary nail, Trauma, Bone

## Abstract

The purpose of this study is to evaluate the efficacy of plate augmentation and hybrid bone grafting for treating atrophic nonunion of the femur with original intramedullary nail retained in situ.In this study, 36 patients with atrophic nonunion of the femur who underwent surgery using the technique of plate augmentation and a hybrid bone grafting while retaining the original intramedullary nail in situ in Xi’an Honghui Hospital from January 2019 to December 2021 were enrolled. 28 patients who met the inclusion and exclusion criteria were ultimately included in the study. These 28 patients, consisting of 20 males and 8 females with a mean age of 38 years, were evaluated based on factors such as operation time, intraoperative blood loss, the average hospitalization days. Additionally, the results and function of these patients were evaluated by union time, Wu’s scores of limb function and incidence of serious complications.All 28 patients achieved bone union at the 12 month follow-up, with an average follow-up time of 14.6 ± 4.2 months.The average operation time was 68.3 ± 11.2 min, and the average intraoperative blood loss was 140 ± 22.6 ml. Patients were hospitalized for an average of 5.8 ± 1.1 days. Full clinical and radiological bone union was achieved on average at 5.1 ± 1.9 months. The mean value of Wu's scores at the 12 month follow-up was significantly higher than before the operation. Limb function was excellent in 27 patients and good in one patient at the 12 month follow-up. However, five patients experienced the lower limb vein thrombosis, including one deep vein thrombosis and four lower limb intermuscular vein thromboses. One patient had a superficial infections of the surgical incision site, while three patients reported pain and numbness where their iliac bone graft was extracted at the 12 month follow-up. The technique of plate augmentation and hybrid bone grafting, combined with retaining the original intramedullary nail in situ has been shown to be a safe, effective, simply and standardizable practice for treating atrophic femoral nonunion with an intact original IMN fixation.

## Introduction

Nonunion of the femur presents a challenge for orthopedic surgeons, especially in atrophic nonunion of the femur which has limited osteogenic potential. Several methods for treating nonunion of long bones have been reported^1–3^, but there is currently no widely recognized gold-standard method for treatment. Intramedullary nail (MN) is the most popular choice for treating a fractured femur, due to its minimally invasive approach and more reasonable biomechanical fixation. However, a higher rate of nonunion were observed in patients treated by IMN fixation. Some studies reported that the rate of nonunion ranges from 0 to 14% in the femur^4–6^. The typical cause of hypertrophic nonunion is thought to be inadequate immobilization of the fracture site. The treatment for hypertrophic nonunion with IMN fixation is commonly accepted and involves surgical intervention to stabilize the fracture site, such as through exchange reaming nailing or augmentative plate fixation. Atrophic nonunion is usually thought to be caused by insufficient blood supply to the fracture site. Although there are several techniques^7–12^ described for managing atrophic nonunion of the femur following intramedullary nailing, the treatment of atrophic nonunion in IMN fixation remains a subject of debate. Factors to consider include whether to keep or replace the IMN, whether to use bone grafting, and whether to choose IMN fixation or plate fixation.

In recent years, our team has utilized a novel technique for treating atrophic nonunion of the femur. This technique involves combining plate augmentation with hybrid bone grafting, while retaining the original intramedullary nail in situ. Our clinical results have shown promise, so the objective of this retrospective study is to assess the effectiveness of this technique in treating atrophic nonunion of the femur.

## Patients and methods

### Study design

In this retrospective study, 36 patients diagnosed atrophic femoral nonunion were enrolled and evaluated from January 2019 to December 2021 in XXX Hospital. Femoral nonunion in all patients was diagnosed by our surgical team based on clinical and radiological evidence. Clinical evidence included persistent pain at the fracture site during the stance phase of walking, while radiological evidence included the absence of progressive callus formation on three monthly follow-up X-rays after 6 months post-surgery.

The inclusion criteria were: (1) nonunion of femoral fracture originally fixed with intramedullary nails, (2) nonunion of diaphyseal femoral fractures from lesser trochanter to 5 cm proximal to adductor tubercle.(3) Femoral nonunion were treated by and plate augmentation and hybrid bone grafting, while retaining IMN in situ.

The exclusion criteria were:(1) hypertrophic nonunion of femur, (2) septic nonunion, (3) younger than 18 years of age, (4) less than 90° range of knee joint motion and (5) a follow-up period of less than 12 months.

**Figure 1:**
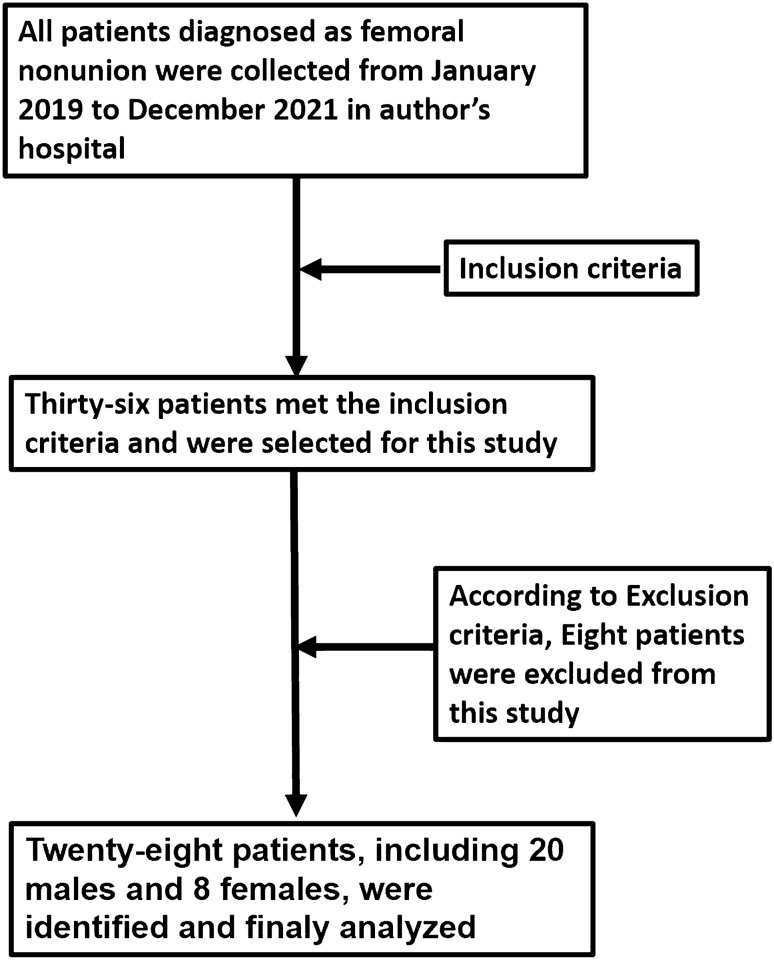
Flow diagram shows how the patients were enrolled in the study.

Thirty-six patients who met the inclusion criteria were included in this study. Two patients were excluded because they were diagnosed with septic nonunion during surgery. Six patients were excluded due to insufficient follow-up time. Finally, 28 patients, including 20 males and 8 females with a average age of 38 years, were identified (Fig. 1). Medical records and radiographs were reviewed from our hospital’s database to identify and analyze details of the initial injury and treatment. All experimental protocols were approved by the Institutional Ethics Committee of the Xi’an Honghui Hospital (No. 2016001, Date March 17, 2019). All methods were carried out in accordance with relevant guidelines and regulations. Informed consent was obtained from all individual participants included in the study. The authors affirm that human research participants provided informed consent for publication of the images in Figs. 3, 4.

### Surgical technique and rehabilitation

All patients underwent surgery using plate augmentation and hybrid bone grafting, while retaining IMN in situ. Hybrid bone grafting involves the use of both structural and granular bone grafts in the same defect to enhance bone healing.

**Figure 2:**
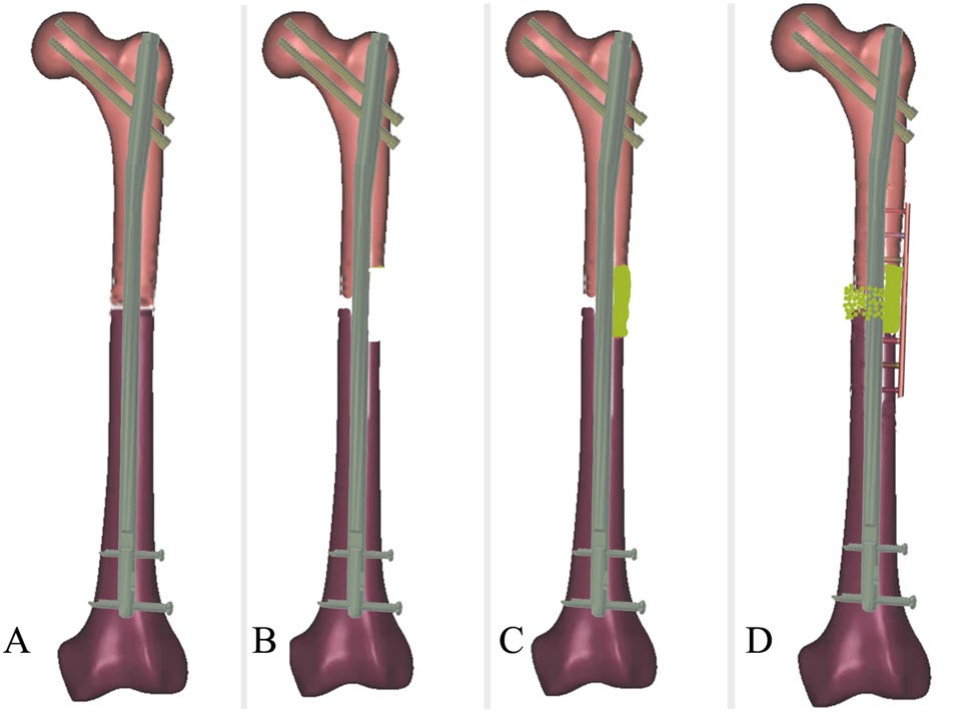
Schematic of surgical technique: (**A**) nonunion of femur fracture, (**B**) first step and second step, (**C**) third step, (**D**) fourth step.

**Figure 3:**
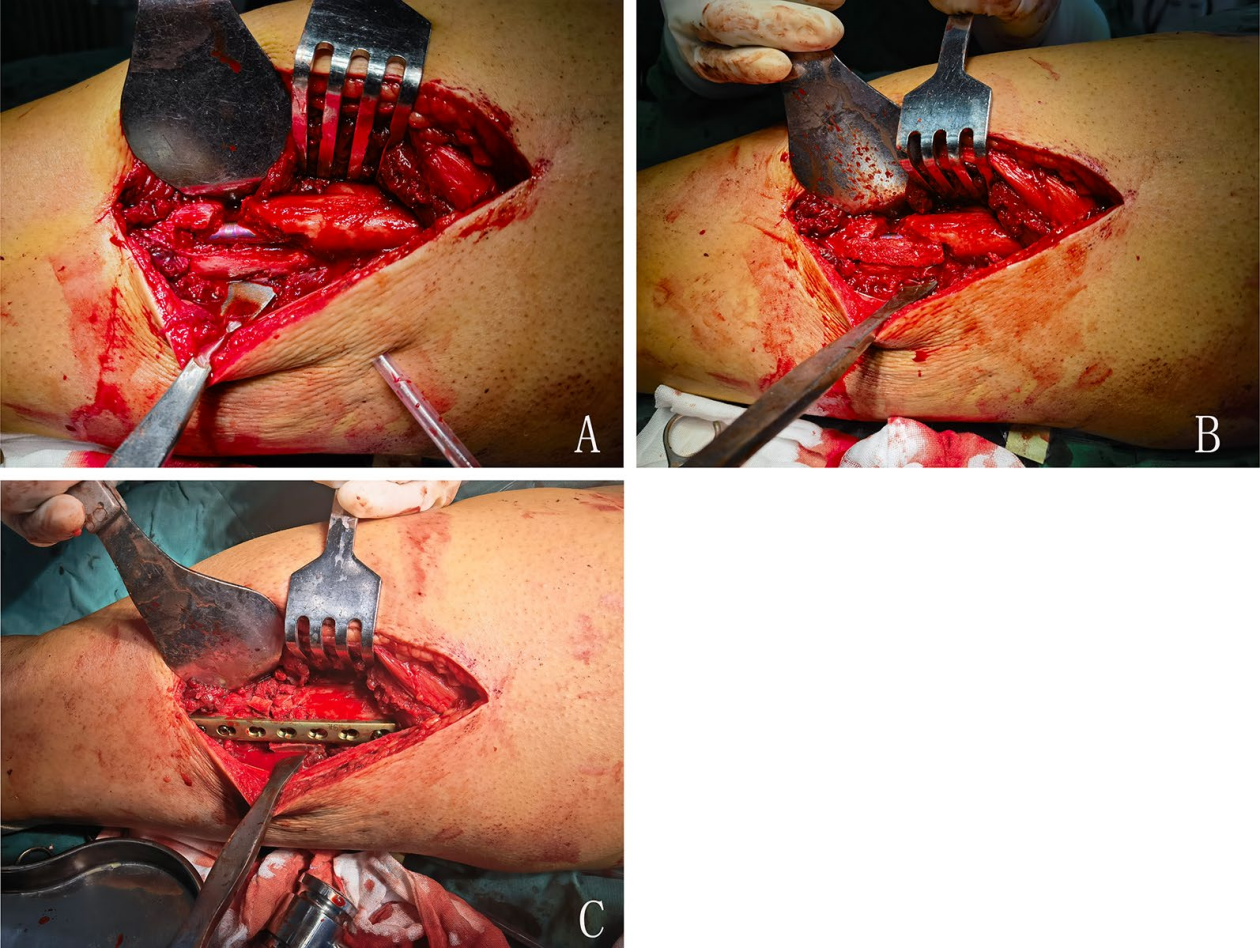
Surgical technique of typical patient. (**A**) All inactivated tissue in nonunion sites was completely debrided. The channel was done by the bone drill and bone knife. (**B**) Structural corticocancellous bone graft obtained from iliac crest was trimed to match the channel, and was impacted in channel to attach the nonunion site. (**C**) Lateral augmentation plate was placed on the surface of the structural bone graft to enhance the stability of fracture fixation. Then, the remaining iliac bone graft was made into about 2–4 mm^3^ bone particles. These cancellous bone particles were filled in the remained gap of nonunion.

All patients were operated on under spinal anesthesia or general anesthesia in the supine position. Surgery was performed using the lateral approach to the femur in the following five steps (Fig. 2):

- First step: The lateral incision of femur was used to expose the nonunion site. All inactivated tissue in nonunion sites was completely debrided, including fibrous tissue and sclerotic bone. In addition, biopsy, antibiogram, and culture were performed for all cases.
- Second step: The technique of lateral femoral cortex drilling was used to remove the 2×1 cm (length×width) lateral femoral cortex at the distal and proximal ends of the fracture nonunion. The drill holes were connected with a bone knife. These cortical bones were moved. A cortical defect in the bone measuring 4×1 cm in length was created.
- Third step: Structural corticocancellous bone grafts were extracted from the iliac crest and trimmed to fit the cortical defect. These were then impacted into the corresponding cortical. The remaining iliac bone graft was broken down into approximately 2–4 mm^3^ granular bones, which were subsequently employed to fill in the remaining gap of nonunion.
- Fourth step: A 3.5 mm LCP was molded and placed on the surface of the structural bone graft on each side of the nonunion with at least three locking holes. Each side of the nonunion was fixed with at least three single cortical locking screws.
- Fifth step: Rinse the surgical site and insert a drainage tube in the deeper layers of the incision. Prophylactic antibiotic therapy was initiated by intravenous administration of second-generation cephalosporins during the surgery and continued for 48 h post-surgery. The irrigation drainage tube was removed 2–4 days after operation. During hospitalization, all patients were administered low molecular weight heparin to prevent deep vein thrombosis. All patients underwent B-ultrasound examination of their lower limb veins on the second day after surgery to determine if there was any presence of deep venous thrombosis. Surgical technique of typical patient wasshown in Fig. 3.

Rehabilitation exercises began on the second day after the operation. These exercises included quadriceps femoris training, ankle pump training, and flexion and extension of nearby joints. If patients experienced pain and apprehension, continuous passive motion (CPM) or rehabilitation physiotherapy was initiated to help with active knee flexion. Starting on the third day after the operation, a walking aid was used to help with walking while bearing partial weight.After 6 weeks, progressive weight bearing was performed based on the clinical and radiological improvements.

### Data collection and analysis

The operation time, intraoperative blood loss, and hospitalization days were extracted from the hospital's patient records to evaluate the level of surgical difficulty and its impact on patients. All patients were followed up at 0, 4, 8, 12, 16, 20, 24, 36, and 52 weeks, and the radiographs and clinical results were evaluated by all authors. Clinical union was defined as the absence of pain during full weight-bearing time, while radiological union was determined based on the modified radiological union scale for tibia (mRUST) score^13^ in anteroposterior and lateral femur radiographs of non-union places. An mRUST score of 10 or more was considered an accurate predictor of healing for metadiaphyseal femur fractures^14^. Outcomes were evaluated based on union time, range of motion in adjacent joints, and medical or surgical complications. CT scans were not routinely employed unless the healing process was difficult to discern from plain radiography.

Wu^15^ scoring system was used to assess the function of the limb. A patient who scores 50 points is considered to have excellent limb function. Patients who score between 30 and 45 points are considered to have good limb function, while those who score less than 15 points are classified as having poor limb function.

### Main complications

Data on complications, including surgical site infections (SSI), deep vein thrombosis (DVT), pulmonary embolism and nonunion, were extracted from patient records. SSI were classified as either superficial surgical site infections or deep surgical site infections according to the definition of the Centers of Disease Control.

### Statistical analyses

All patient data was recorded using Microsoft Excel. The average time of follow-up, union time, patient age, blood loss, hospitalization days, etc., were analyzeded using SPSS 18.0. A sample t-test was used to analyze statistical significance, with the significance level set at a p value less than 0.05.

## Results

All 28 patients, including 20 males and 8 females with a average age of 38 ± 4.9 years (range 22–56 years), were evaluated in our study. Among them, 26 patients had femoral shaft fractures and 2 patients had femoral subtrochanteric fractures. The average follow-up time was 14.6 ± 4.2 months (range 12–33 months). Average interval time since primary surgery to surgery using plate augmentation combined with a hybrid bone grafting was 13.6 ± 2.2 months (range 9–17 months). The average operation time was 68.3 ± 11.2 min (range 52–98 min) and average intraoperative blood loss was 140 ± 22.6 ml (range 100–220). The average hospitalization days was 5.8 ± 1.1 days (range 4–9). The main characteristics of the patients were show in Table 1.

**Table 1:** Main characteristics of the patients.

Characteristics	Patients (28)
Age (years)	38 ± 4.9
Sex	
Male	20
Female	8
Nonunion site	
Femoral shaft nonunion	26
Femoral subtrochanteric nonunion	2
Follow-up time (months)	14.6 ± 4.2
Interval time of revision surgery (months)	13.6 ± 2.2
Operation time (min)	68.3 ± 11.2
Intraoperative blood loss (ml)	140 ± 22.6
Hospitalization days (days)	5.8 ± 1.1

### Clinical outcomes

**Figure 4:**
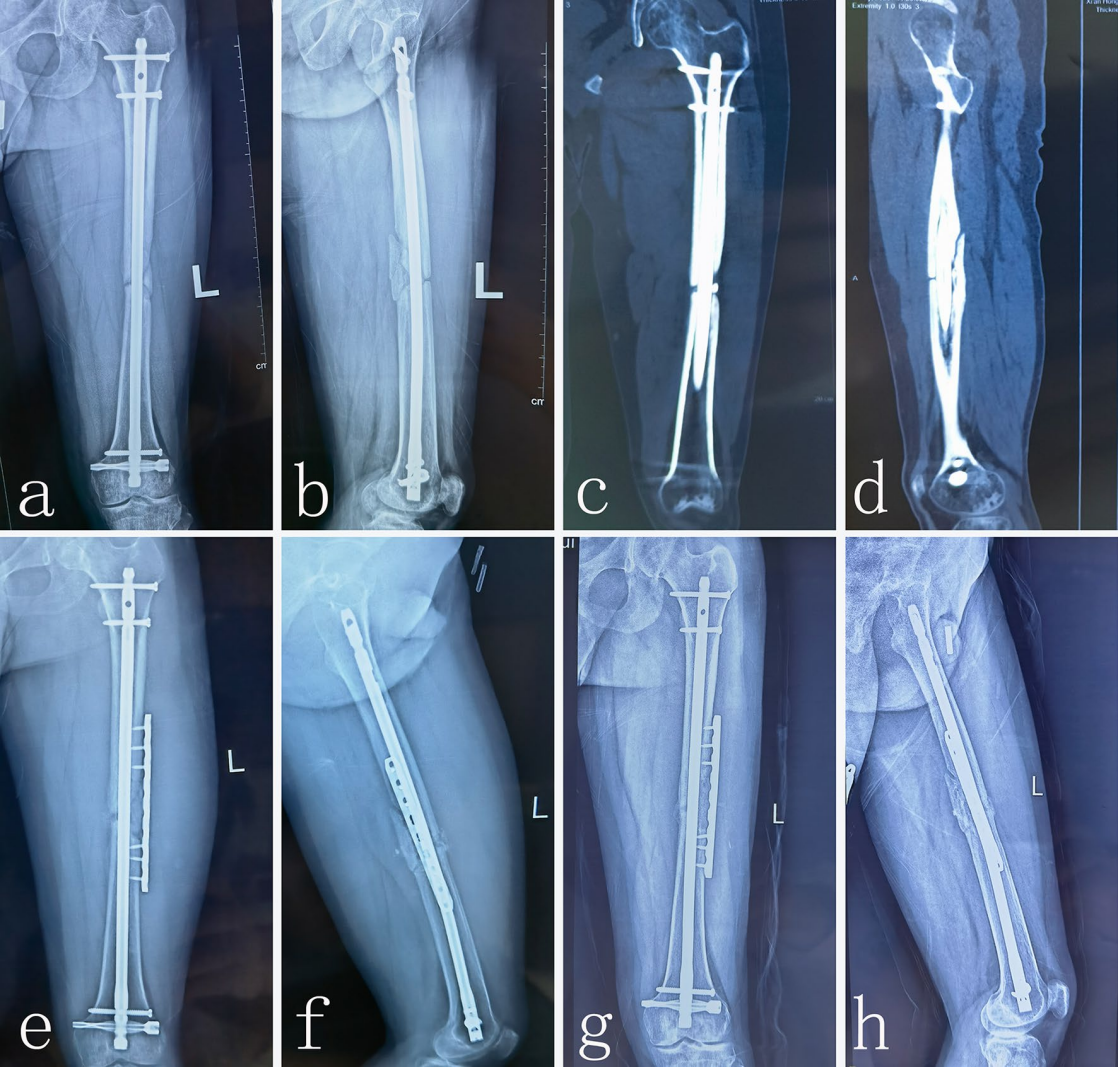
Radiological image of typical patient with nonunion of femoral shaft. The preoperative anteroposterior X-ray (**a**) and The preoperative lateral X-ray (**b**) showed that the atrophic nonunion of femur shaft was clear. The coronal and sagittal 2d-CT images (**c**,**d**) further demonstrate the atrophic nonunion of femur shaft. An anteroposterior X-ray (**e**) and lateral X-ray (**f**) at the 2th day after the operation showed that fracture lines were clear. An anteroposterior X-ray (**g**) and lateral X-ray (**h**) at the 6th months after the operation showed that fracture lines were blurred and new callus forms at the site of the fracture. The result of X-ray shows the atrophic nonunion of femur shaft had healed well.

All 28 patients achieved bone union after one operation using the technique of plate augmentation and hybrid bone grafting while retaining IMN in situ. The average bone union time to full clinical and radiological union was 5.1 ± 1.9 months (range 4–7 months). Photographs and X-ray images of typical patients are shown in Fig. 4. The average value of Wu’s scores was 13 points before the operation. In the 12 month follow-up, the average value of Wu’s scores was 47 points. This was a significant increase compared to preoperation scores. In 12 months follow-up, 27 patients had excellent limb function and one patient had good limb function. The main clinical outcomes were show in Table 2.

**Table 2:** Clinical outcomes and main complications.

Clinical outcomes and main complications	Patients (28)
Time to union (months)	5.1 ± 1.9
Vein thrombosis	4
SSI	1
Superficial SSI	1
Deep SSI	0
Pain or numbness in iliac bone graft site	3
The value of Wu’s scores*	
Preoperation	13 ± 2.2
Postoperation	47 ± 6.4

*Shows that the mean value of Wu’s scores of postoperation is significantly higher than that of preoperation (p <0.05).

### Complications

Five patients had suffered from lower limb vein thrombosis including one DVT and four lower limb intermuscular vein thrombosis. All patients with lower limb vein thrombosis had not the clinical symptoms and were treated successfully by an expert medical team. One patient had suffered from superficial infections of surgical incision. No deep infection of surgical site was observed in 12 months follow-up. Three patients have complained of pain and numbness where their iliac bone graft was extracted in 12 months follow-up. No implants broken including plate, screws, lock-screws and IMN were observed in 12 months follow-up. The main complications were show in Table 2.

## Discussion

The technique of intramedullary nailing is a standard method for treating femur fractures. In clinical practice, some patients who undergo IMN fixation may experience femoral nonunion. Several surgical methods have been reported for treating nonunion in patients who have undergone IMN fixation, such as early dynamization of interlocking intramedullary nails, exchange reaming nailing, augmentative plate fixation, autologous bone grafting through channels combined with double-plate fixation, and others^7–11^ . There is no widely recognized gold-standard method for the treatment of long bone nonunion. In clinical practice, we found that the nonunion of femur is divided into two types: intact IMN fixation and broken IMN fixation. For patients with a broken IMN fixation, the consistent treatment approach is to remove the broken IMN and instead use reaming nailing or plate fixation. For patients with an intact IMN fixation, there is controversy as to whether or not to remove the original intramedullary nail.

In our study, for the patients with an intact original IMN fixation, the technique of plate augmentation and hybrid bone grafting was used to treat the atrophic femoral nonunion with original intramedullary nail retained in situ. The technique provided sufficient stability and biological stimulation for successful bone healing. Atrophic nonunions of the femur are often challenging to treat because of the limited blood supply in the area, leading to poor bone healing. Creating a cortical defect in the region of the nonunion is similar to a new fracture and helps promote sufficient blood supply, osteoblasts, and cytokines. The retained intramedullary nail provides stability for the fracture site, while the augmentation plate adds additional resistance to bending and rotational forces. The hybrid bone grafting technique provides both structural support and biological stimulation for bone regeneration. The cancellous structural graft acts as a scaffold for bone regeneration, while the granule bone graft helps recruit bone-forming cells and aids in bone healing.

Our study results showed that the mean intraoperative blood loss was 140 ml (range 100–220), the mean operating time was 68 min (range 52–98), and the mean radiological bony union time was 5.1 months (range 4–7). In the study by Choi and Kim^16^, they reported on 15 patients with femoral nonunion after interlocking intramedullary nailing who were treated with plate augmentation and bone grafting with the nail in situ. Their results showed a mean intraoperative blood loss of 237 ml (range 150–300) and a mean radiological bony union time of 7.2 months (range 5–11). In Mittal’s study^17^, augmentation plating and bone grafting were performed in eighteen patients who retained the previous nail in situ, while in three other patients, the broken nail was exchanged for the same size unreamed nail and a similar procedure was carried out. Their results showed that the average operating time was 70 min (range 50–85) and all patients achieved bony union in a mean time of 6 months (range 4–8). In our previous study^10^, we reported on the treatment of femoral nonunion using the technique of “autologous bone grafting through channels” combined with double-plate fixation. Our results showed a mean radiological union time of 7.6 months (range 4–9). Compared with previous studies^9,10,13,16,17^, our study demonstrated shorter operation time, less intraoperative blood loss, and shorter bone union times.

In our study, an augmentation plate was chosen to fix the atrophic nonunion due to three factors. First, the fibrous tissue and sclerotic bone at the nonunion site could be removed and the cancellous bone could be grafted through the incision of the augmentation plate. Second, the intact original IMN fixation could still provide sufficient mechanical stability and avoid additional financial burden and surgical damage. Last, exchanged reamed nailing is ill-advised for atrophic nonunion in previous reports^18^. Exchange nailing is known to be the most acceptable method of treatment for femoral non-union but with varying successful results^19,20^. Studies have shown that using a larger nail, which reams the canal by an extra 2–3 mm, can improve mechanical stability by increasing the length of the isthmus. This allows for better contact between the nail and the bone. Additionally, the marrow debris after reaming acts as bone graft. Exchanged reamed nail is useful in hypertrophic non-unions where nonunion is mainly because of mechanical failures. Augmentation plating is also an effective treatment with no absolute or relative contraindications for any type of non-union or any fracture geometry. Several studies have reported 100% successful outcomes when using augmentation plating to address this challenging issue^21–23^.

Some studies^16,17,23–26^ have reported that the technique of plate augmentation, bone grafting and intramedullary nail retained in situ yields good results for the nonunion of the femur. However, our study differs significantly from these studies in terms of pre-treatment of the bone grafting site and bone grafting techniques. In some studies^24,25^, the nonunion site was treated by removing the fibrous tissue and sclerotic bone. In clinic practice, removing the sclerotic bone or cortical bone with insufficient vitality is very difficult to be removed thoroughly. In our experience, extensive removal of inactive cortical bone could refresh the nonunion site, resulting in large bone defects. Other studies^17,27,28^ have used a decortication technique to remove fibrous tissue and sclerotic bone at the nonunion site, but this method lacks clear standardization and relies mainly on a surgeon's personal experience. To overcome these challenges and avoid large bone defects, our study utilized a hybrid bone grafting technique that simplifies the process and provides greater standardization.

## Conclusion

The treatment of atrophic femoral nonunion is very challenging and not typically standard practice. The technique of plate augmentation and hybrid bone grafting, combined with retaining the original intramedullary nail in situ has been shown to be a safe, effective, simply and standardizable practice for treating atrophic femoral nonunion with an intact original IMN fixation. Our results indicate that this technique has a shorter operation time, less intraoperative blood loss, and a shorter bone union time. Nevertheless, a larger prospective controlled trial will be necessary to further study these outcomes.

## Data Availability

The datasets generated during and/or analysed during the current study are available from the corresponding author on reasonable request.
